# DNA hypomethylation upregulates expression of the *MGAT3* gene in HepG2 cells and leads to changes in *N*-glycosylation of secreted glycoproteins

**DOI:** 10.1038/srep24363

**Published:** 2016-04-13

**Authors:** Marija Klasić, Jasminka Krištić, Petra Korać, Tomislav Horvat, Dora Markulin, Aleksandar Vojta, Karli R. Reiding, Manfred Wuhrer, Gordan Lauc, Vlatka Zoldoš

**Affiliations:** 1 University of Zagreb Faculty of Science, Zagreb, Croatia; 2 Genos Glycoscience Research Laboratory, Zagreb, Croatia; 3 Center for Proteomics and Metabolomics, Leiden University Medical Center, Leiden, The Netherlands; 4 Division of BioAnalytical Chemistry, VU University Amsterdam, Amsterdam, The Netherlands; 5 University of Zagreb Faculty of Pharmacy and Biochemistry, Zagreb, Croatia

## Abstract

Changes in *N*-glycosylation of plasma proteins are observed in many types of cancer, nevertheless, few studies suggest the exact mechanism involved in aberrant protein glycosylation. Here we studied the impact of DNA methylation on the *N*-glycome in the secretome of the HepG2 cell line derived from hepatocellular carcinoma (HCC). Since the majority of plasma glycoproteins originate from the liver, the HepG2 cells represent a good model for glycosylation changes in HCC that are detectable in blood, which is an easily accessible analytic material in a clinical setting. Two different concentrations of 5-aza-2′-deoxycytidine (5-aza-2dC) differentially affected global genome methylation and induced different glycan changes. Around twenty percent of 84 glyco-genes analysed changed expression level after the 5-aza-2dC treatment as a result of global genome hypomethylation. A correlation study between the changes in glyco-gene expression and the HepG2 glycosylation profile suggests that the *MGAT3* gene might be responsible for the glycan changes consistently induced by both doses of 5-aza-2dC. Core-fucosylated tetra-antennary structures were decreased in quantity likely as a result of hypomethylated *MGAT3* gene promoter followed by increased expression of this gene.

Glycosylation is an important post-translational modification that can significantly contribute to variability in protein structures^1^,^2^. While many protein modifications, such as phosphorylation, generally function as on/off switches, glycosylation is more diverse. A number of different glycans can be attached to the same glycosylation site, resulting in alteration of both glycoprotein structure and function. Therefore, glycosylation is a vital functional and regulatory modification of proteins and almost all membrane and secreted proteins are covalently modified by complex oligosaccharides. Glycans attached to proteins or lipids define the large part of the surface of mammalian cells, making glycans the major determinants in cellular interaction and communication^3^. Moreover, glycans integrate genetic and environmental factors, and are thus closely associated with complex diseases^4^.

Glycans are synthesized in a complex biochemical chain of reactions, involving many enzymes and other proteins^5^. Classical glyco-genes (coding for glycosidases, glycosyltransferases, *etc*.) represent only a part of this large metabolic pathway that also includes numerous transcription factors, transport proteins, ion channels, Golgi organizers, *etc*. Current studies indicate that glycosylation pathways include at least three to four times more genes than the currently listed 800 glyco-genes, suggesting that more than 10% of the genome may be involved in glycosylation^6^,^7^,^8^. Regulation of glycosylation seems to be very complex and still not fully understood–it is under the influence of genetic and epigenetic factors as well as various internal and external environmental factors^2^,^4^,^5^,^9^,^10^,^11^. Regulatory mechanisms may induce changes in the activity, relative abundance and/or localization of any of the enzymes involved in glycan biosynthesis affecting the final structure of a glycan. Nevertheless, little is known about mechanisms leading to extensive changes in the plasma glycome composition in disease. In fact, virtually every human disease is associated with glycosylation changes^12^,^13^,^14^. Alternative glycosylation represents a universal feature of malignant transformation and tumour progression^13^,^15^. The role of glycosylation is also pronounced in the context of tumour metastasis^16^.

Protein glycosylation is under strong genetic regulation however, when homeostasis is disturbed, for example during acute systemic inflammation, composition of both total plasma and IgG glycomes can change rapidly^9^,17,^18^. Interestingly, upon restoration of homeostasis, the glycome composition returns to the initial state that is characteristic of an individual^19^. Therefore, protein glycosylation appears to be dynamic and flexible during the lifetime of an adult individual and is largely influenced by environment. Epigenetic mechanisms are established mediators between environment, genotype and the final phenotype^20^. We and others have shown that the glycosylation patterns of cell membrane proteins are regulated by both DNA methylation and histone acetylation and that this regulation is stable^21^,^22^,^23^,^24^. In addition, Saldova *et al.*^25^ have shown that the glycosylation of secreted proteins is epigenetically regulated. Recent data also points to a role of micro RNAs (miRNAs) in modulating expression level of glyco-genes with biological consequences including promotion of tumour metastasis^26^,^27^,^28^. Furthermore, the aberrant epigenetic regulation of specific glyco-genes was shown in many cancers^15^,29,^30^.

Since majority of plasma proteins are produced by hepatocytes, and blood is the most convenient sample to search for diagnostic and prognostic markers in many diseases, we studied the impact of genome DNA hypomethylation on protein glycosylation in the hepatocellular carcinoma (HCC) cell line, HepG2. We showed that 18 out of 84 glyco-genes changed their expression level after the treatment with the inhibitor of DNA methyltransferases, 5-aza-2′-deoxycytidine (5-aza-2dC), and that this change was correlated with changes in the composition of *N*-glycome from the HepG2 secretome. We also propose that aberrant expression of the *MGAT3* gene as a result of DNA hypomethylation is the mechanisms leading to the aberrant glyco-phenotype characteristic of HCC.

## Results

### Two different doses of 5-aza-2dC differently affect DNA methylation and the cell cycle of HepG2 cells

**Figure 1:**
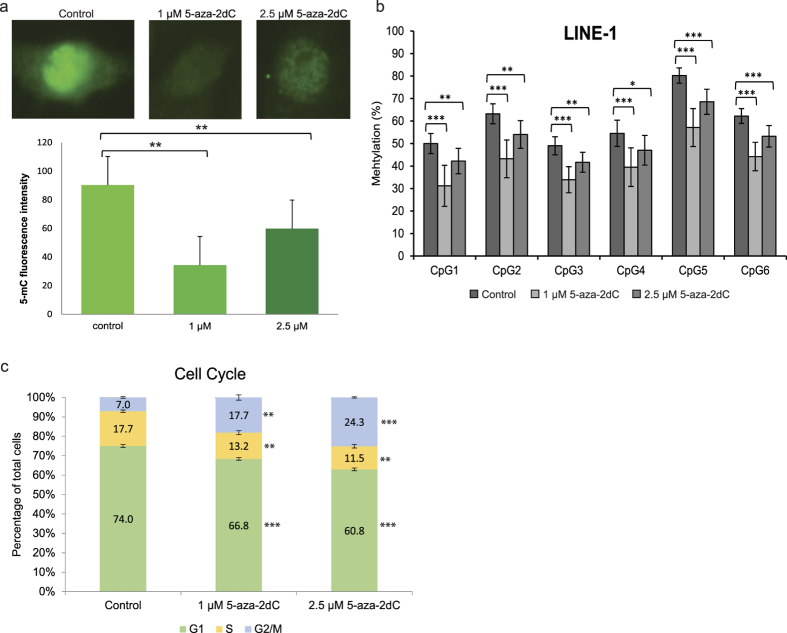
(**a**) Immunofluorescence using anti-5mC antibody and integrated intensity fluorescence analysis revealed a decrease in global genome CpG methylation in HepG2 cells following treatment with 1 μM and 2.5 μM 5-aza-2dC when compared to the control group (p < 0.005). Error bars show standard deviation of fluorescence intensity in nuclei of each group. (**b**) Methylation levels at six CpG sites in the LINE-1 element were significantly decreased in treated groups as compared to control. Error bars show standard deviation between biological replicates. Asterisks in the stacked bars indicate statistical significance: *p < 0.05; **p < 0.01; ***p < 0.001. (**c**) Cell cycle profiles for untreated cells and cells treated with 1 μM and 2.5 μM 5-aza-2dC 5-aza-2dC show statistically significant differences to the level of *p* < 0.01. Both concentrations of 5-aza-2dC induced a decrease in the proportion of cells in G1 and S-phase, and an increase in the proportion of cells in G2/M phase. The higher the concentration of 5-aza-2dC, the lower was the proportion of the cells in G1 phase and the higher was the proportion of the cells stalled in G2/M phase (*p* < 0.01). Error bars show standard deviation between biological replicates.

To study the impact of epigenetic changes on the glycan profile of HepG2 secretome, cells were treated with the DNA methyltransferase (DNMT) inhibitor 5-aza-2dC. Prior to *N*-glycome analysis, the efficiency of the treatment was checked by immunofluorescence (IF) using anti-5mC antibody (Fig. 1a), and by pyrosequencing of the LINE-1 elements (Fig. 1b). Pyrosequencing analysis of the LINE-1 elements showed a significant decrease (ranging from *p* = 0.0001 to *p* = 0.001) in CpG methylation after the treatment with 1 μM 5-aza-2dC, indicating global genome hypomethylation (Fig. 1b). Interestingly, the decrease in the methylation level of LINE-1 elements after the treatment with 2.5 μM 5-aza-2dC was not as extensive as after the 1 μM 5-aza-2dC treatment. The same trend of DNA methylation change was noticed after the visual inspection of 5mC IF signal using fluorescence microscopy (Fig. 1a), which was confirmed by integrated intensity fluorescence analysis–a slightly higher decrease in the level of CpG methylation was noticed after 1 μM than after 2.5 μM 5-aza-2dC treatment.

In order to see the effect of 5-aza-2dC on the HepG2 cell cycle progression, we have analysed untreated and treated cells by flow cytometry (Fig. 1c, Suppl. Fig. 1). We observed statistically significant changes (*p* < 0.01) between untreated cells and those treated with either 1 μM or 2.5 μM 5-aza-2dC for all phases of the cell cycle. Following treatment, we observed fewer cells in G1 and S phases and more cells in the G2/M phase with the effect increasing along with the concentration of 5-aza-2dC.

### *N*-glycome of HepG2 secretome and expression level of glyco-genes change following the treatment with 5-aza-2dC

**Figure 2:**
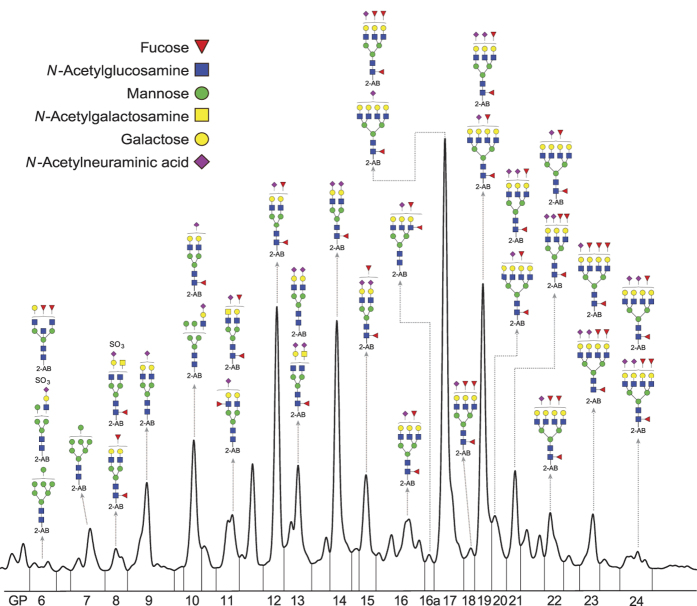
UPLC analysis of the *N-*glycome of HepG2 secretome. The *N*-glycome from secretome of untreated HepG2 cells was separated into 20 chromatographic peaks (GP6-GP24) by hydrophilic interaction chromatography (HILIC). The structures of the *N*-glycans present in each peak were characterized by fractionation and subsequent MALDI-TOF-MS and LC-ESI-MS(/MS) analysis (see Suppl. Table 1). GP = glycan peak.

**Figure 3:**
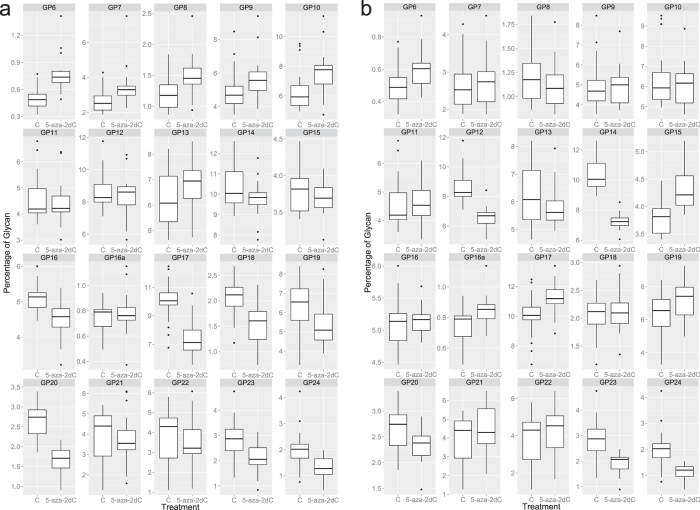
Contribution of individual glycan structures to the total *N*-glycome of the HepG2 secretome as measured after the treatment with the DNA methylation inhibitor 5-aza-2dC. (**a**) The glycan structures under the peaks GP6, GP7, GP16, GP17, GP18 and GP20 were significantly changed following the treatment with 1 μM 5-aza-2dC. (**b**) The glycan structures under the peaks GP12, GP14, GP15 GP23 and GP24 were significantly changed following the treatment with 2.5 μM 5-aza-2dC. *p* < 0.05; GP = glycan peak.

The glycoproteins secreted from HepG2 cells were collected after 72 hours of the 5-aza-2dC treatment. *N*-glycans were released from proteins and analysed by HILIC-UPLC (Fig. 2). The composition of glycan structures in each UPLC peak was determined by MALDI-TOF-MS and LC-ESI-MS(/MS) (Supplementary Table 1). Treatment with 5-aza-2dC induced significant changes in the *N*-glycome composition of secreted proteins (Table 1): six glycan groups were significantly changed in quantity following the 1 μM 5-aza-2dC treatment and these are GP6 (p = 0.006); GP7 (p = 0.018); GP16 (p = 0.002); GP17 (p = 0.014); GP18 (p = 0.009) and GP20 (p = 0.002) (Fig. 3a, Supplementary Table 1). These changes included increase in oligomannose structures without core-fucose and decrease in complex glycan structures with more than two branches (i.e. tri- and tetraanntenary glycans). The 2.5 μM 5-aza-2dC treatment caused changes in quantity of glycan structures corresponding to GP12 (p = 0.014), GP14 (p = 0.005), GP15 (p = 0.041), GP23 (0.029) and GP24 (p = 0.009) groups (Fig. 3b, Supplementary Table 1). Although the glycan changes induced by two different concentrations of 5-aza-2dC differed in general, the decrease in more complex branched glycans was the main feature of the both 5-aza-2dC treatments.

**Figure 4:**
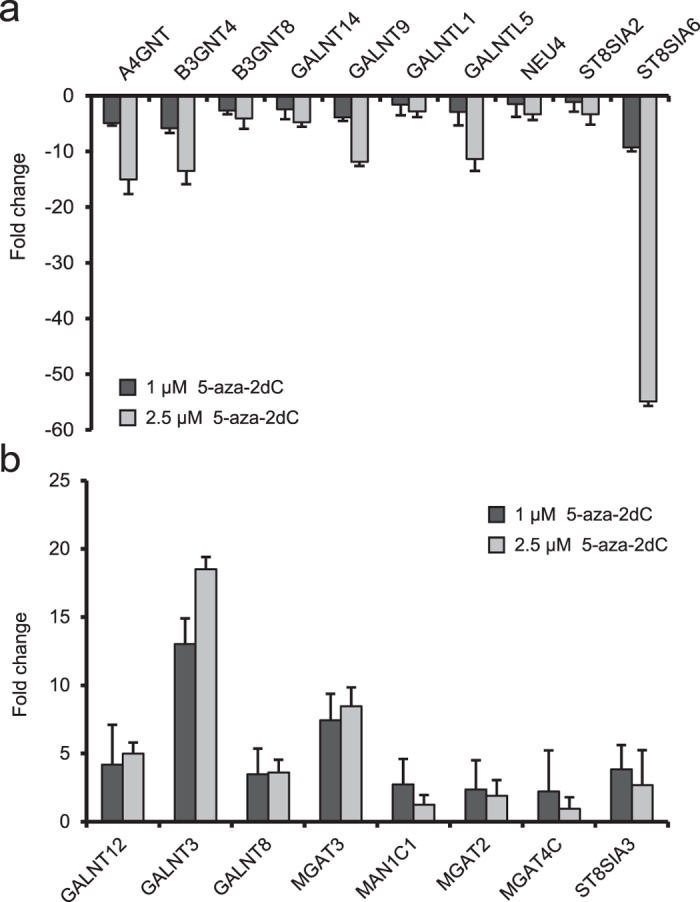
Gene expression analysis performed using Glycosylation RT^2^ Profiler PCR Array revealed glyco-genes that were down-regulated (**a**) or up-regulated (**b**) following the treatment with 1 μM and 2.5 μM 5-aza-2dC, respectively. Error bars indicate standard deviation between biological replicates.

**Figure 5:**
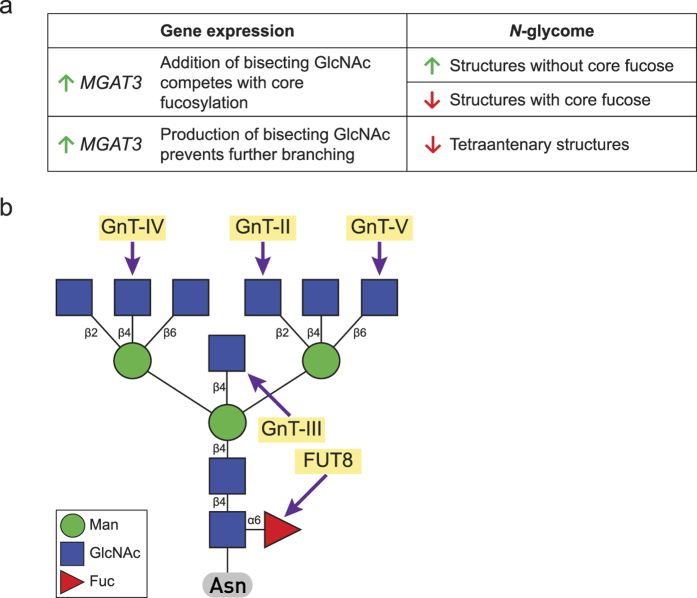
(**a**) The consistent changes in the glycome of HepG2 secretome was a decrease in the quantity of the glycans with core fucose and tetra-antennary glycans, which could be explained by elevated *MGAT3* gene expression. (**b**) Addition of a bisecting GlcNAc by the catalytic activity of GnT-III (the *MGAT3* gene) inhibits the elongation of a glycan structure by inhibiting GnT-IV (the *MGAT4* gene), GnT-V (the *MGAT5* gene) and Fut8 (the *FUT8* gene).

Effects of 5-aza-2dC treatment on the expression of 84 glyco-genes were analysed using the Glycosylation RT^2^ Profiler PCR Array, which includes the main glycosyltransferases and glycosidases involved in both *N*- and *O*-glycosylation. Considerable difference in the expression level of 18 genes was detected, suggesting that approximately 20% of the glyco-genes were affected by the global genome hypomethylation (Fig. 4, Supplementary Table 2). Correlation analysis between the changes in the *N*-glycome composition of HepG2 secretome and the changes in glyco-gene expression did not reveal a one-to-one link. Nevertheless, the most prominent glycan change that reproducibly appeared following the treatment with both concentrations of 5-aza-2dC–a decrease of highly branched *N*-glycans (Fig. 3a,b)–could be potentially linked to the increased expression of the *MGAT3* gene (Fig. 5). An increase in structures without core-fucose observed after the treatment with 1 μM 5-aza-2dC (Fig. 3a) can also be explained with increased *MGAT3* expression. Namely, GlcNAc transferase encoded by the *MGAT3* gene is responsible for addition of bisecting GlcNAc (*N*-acetylglucosamine) onto the 3-mannose core, which is known to prevent further addition of core fucose and branching^31^.

### Alteration of transcription and methylation levels of the *MGAT3* gene associates with changes in particular glycan structures

**Figure 6:**
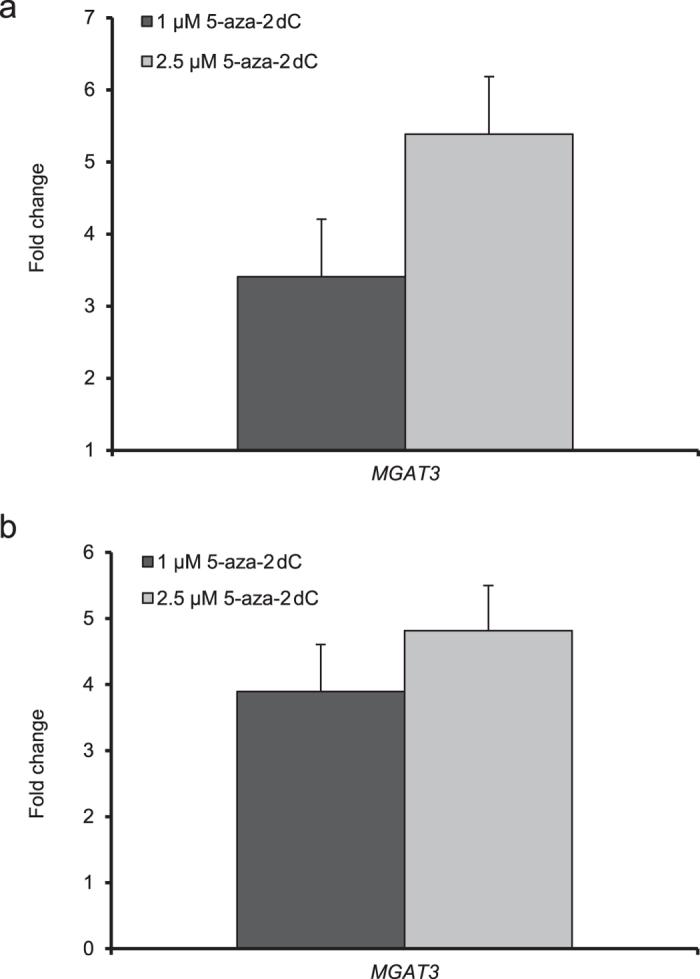
The expression level of the *MGAT3* gene as measured following 5-aza-2dC treatment. (**a**) Experiment 1 showed 3.4-fold and 5.4-fold up-regulation of the *MGAT3* expression (*p* < 0.05) after the treatment with 1 μM and 2.5 μM 5-aza-2dC, respectively. (**b**) Experiment 2 showed 3.9-fold and 4.8-fold up-regulation of the *MGAT3* expression (*p* < 0.01) after the treatment with 1 μM and 2.5 μM 5-aza-2dC, respectively. The experiments were done in five biological replicates. Error bars indicate standard deviation between biological replicates.

Initial results of the changed expression of glyco-genes from the Glycosylation RT^2^ Profiler PCR Array and the correlation analysis with the glycan changes picked out the *MGAT3* gene as the one that could explain the most consistent changes in the *N*-glycome of HepG2 cells (i.e. decrease in quantity of core-fucosylated tetra-antennary structures) after 5-aza-2dC treatment. Therefore, we validated expression and methylation level of this gene before and after the 5-aza-2dC treatment using qPCR with specific TaqMan probes and using pyrosequencing of bisulphite converted DNA. Two experimental replicates were done. Replication study 1 showed 3.4-fold and 5.4-fold upregulation of *MGAT3* expression (p = 0.028; p = 0.014) after the treatment with 1 μM and 2.5 μM 5-aza-2dC, respectively (Fig. 6a). In the replication study 2, the *MGAT3* gene expression was 3.9-fold and 4.8-fold increased compared to the control (p = 0.008; p = 0.009) after the cells were treated with 1 μM and 2.5 μM 5-aza-2dC, respectively (Fig. 6b).

**Figure 7:**
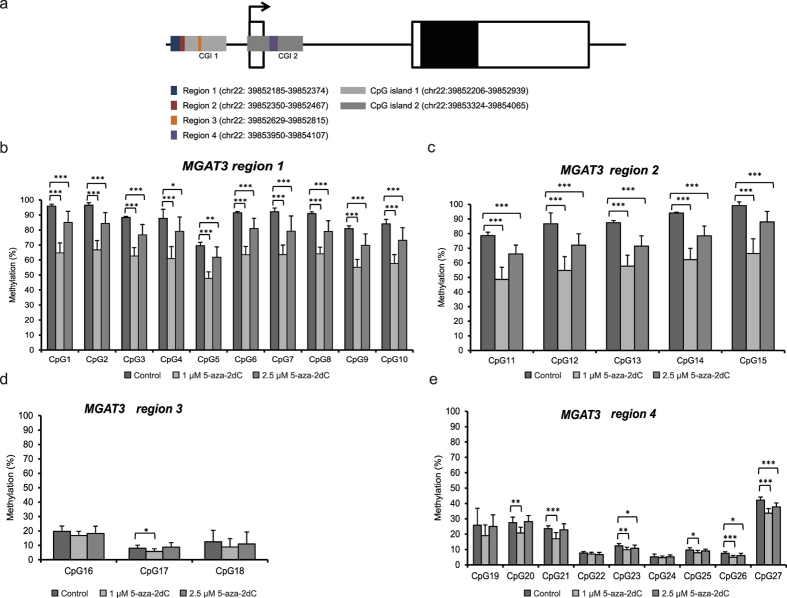
Methylation levels in the promoter/first intron of the *MGAT3* gene as measured after 5-aza-2dC treatment. (**a**) Schematic representation of the *MGAT3* gene. The promoter (1,000 bp upstream from the transcription start site, indicated by an arrow) has one CpG island (CpG island 1), which is 734 bp long. Second CpG island (CpG island 2, 742 bp long) includes the first exon and intron. White boxes represent untranslated (UTR) exons, and the black box stands for coding exon. The location of the four regions, containing CpG sites analysed for methylation level, is represented as coloured boxes. (**b**) Decrease in methylation level at 10 CpG sites in the region 1 (*p*-values ranging from *p* < 0.001 to *p* < 0.005). (**c**) Decrease in methylation level at 5 CpG sites within the region 2 (*p* < 0.001). (**d**) Within the region 3, only the site CpG22 shows statistically significant decrease in methylation level following 1 μM 5-aza-2dC treatment (p < 0.05); (**e**) Decrease in methylation level in the region 4 (*p*-values ranging from *p* < 0.001 to *p* < 0.05). Asterisks in the stacked bar graphs indicate statistical significance: *p < 0.05; **p < 0.01; ***p < 0.001.

We also analysed methylation at specific CpG sites within the *MGAT3* gene in order to see if the gene expression change could be associated to a local change in DNA methylation. Our specific assays were designed to cover 32 CpG sites (in total) located within the *MGAT3* promoter (two CpG islands) and the first intron (Fig. 7a) in order to identify the elements that might be involved in regulation of the *MGAT3* gene expression. While the methylation levels at 5 CpG sites within the region 5 were low in both groups (less than 10%, Suppl. Fig. 2), there was a significant decrease in the methylation levels at all 10 CpG sites within the region 1, five CpG sites within the region 2, and few of the CpG sites within the region 4 after the treatments with both concentrations of 5-aza-2dC (Fig. 7). A decrease in methylation level at 6 out of 9 CpG sites was statistically significant following 1 μM 5-aza-2dC treatment (Fig. 7e). For all CpG sites, which showed changed methylation levels compared to the control, the same trend of a decrease was noticed–the methylation level was less decreased following 2.5 μM 5-aza-2dC treatment than following 1 μM 5-aza-2dC treatment (Fig. 7b–e).

### Meta-analysis of *MGAT3* promoter methylation and gene expression level

**Figure 8:**
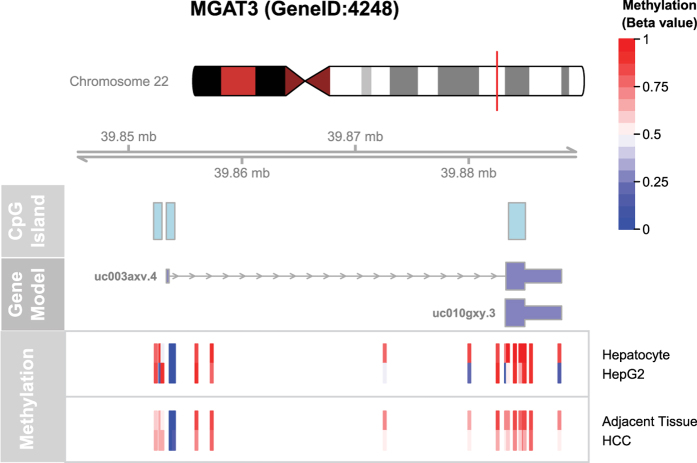
Methylation level of the *MGAT3* promoter region in HepG2 cell line and in HCC. Gene model and coordinates from the hg19 human genome assembly are shown, with location of CpG islands highlighted on a separate track. Methylation of the promoter region covered by the Illumina 450k platform is shown in colors corresponding to the methylation level. Differential methylation can be observed between probes located within CpG islands, with HepG2 cell line being hypomethylated in comparison to normal hepatocytes, as well as HCC when compared to adjacent tissue.

Meta-analysis of the *MGAT3* promoter methylation revealed hypomethylation in hepatocellular carcinoma (HCC) compared to adjacent non-tumor tissue. Similar trend of DNA methylation decrease in the *MGAT3* promoter could be observed in HepG2 cell line when compared with normal hepatocytes (Fig. 8). In addition, the promoter methylation changes in HCC were in line with the higher expression of the *MGAT3* in HCC tissue, which was 128% or 126% of the expression level in the paired adjacent tissue (GSE60502) or healthy liver (GSE62232), respectively. Both changes in the expression level were highly significant (*p*-values 2.5 × 10^−6^ and 4.9 × 10^−6^).

## Discussion

Changes in plasma protein glycosylation have been reported in various types of cancer as well as in numerous other complex diseases^12^,^13^,^32^,^33^,^34^,^35^. Many of these studies report *N*-glycans as markers with prognostic, diagnostic or recurrence value, but only a few of them suggest how glycans contribute to tumour/disease development and progression^35^,^36^,^37^. In this work, we investigated transcriptional profiling of glyco-genes in a HCC-related cell line (HepG2) and correlated the results with the *N*-linked glycans on glycoproteins secreted from HepG2 cells after the treatment with 5-aza-2dC, the inhibitor of DNA methyltransferase 1 (DNMT1)^38^. We found that around 20% of the glyco-genes were differentially expressed as a result of the global genome hypomethylation. Even though treatments with two different concentrations of 5-aza-2dC resulted in change of different glycan groups, the glycan structures that were found consistently changed in quantity in HepG2 cells after both treatments could be explained with the increased expression of the *MGAT3* gene, due to demethylation at specific CpG sites.

Different concentrations of the DNA methylation inhibitor caused nonlinear changes in global genome methylation. Contrary to our expectation that higher concentration of 5-aza-2dC would cause more pronounced genome hypomethylation, we observed a different trend. The decrease in global genome methylation was more extensive after the treatment with 1 μM then after the treatment with 2.5 μM 5-aza-2dC. The same trend of CpG hypomethylation was observed in LINE-1 elements, as well as in specific CpG sites within promoter and the first intron of the *MGAT3* gene. The analysis of the cell cycle profile showed that 5-aza-2dC induced changes in cell cycle distribution. There was a tendency of cells to accumulate in the G2/M phase, which could eventually lead to the cell cycle arrest. This is in line with previously published data on effects of the same DNA methylation inhibitor on mouse embryonic fibroblasts (MEFs)^39^. Our results of the cell cycle analysis provide a possible explanation for different effect of the two different doses of 5-aza-2dC on global genome and specific promoter methylation observed in our experiments. As a higher concentration of 5-aza-2dC caused accumulation of HepG2 cells in G2/M phase, thus reducing the number of cells which are cycling and replicating, the effect of this dose on the global genome hypomethylation would therefore appear less pronounced. More importantly, differential effects of the two different concentrations of 5-aza-2dC on HepG2 cell cycle progression were consistently recorded. A statistically significant difference in percentage of cells in the G1 phase and the G2/M phase was recorded after the 1 μM and 2.5 μM 5-aza-2dC treatment, which is in concordance with the observed difference of 5-aza-2dC effect on the level of DNA methylation.

We have found that different glycan structures change in quantity within the HepG2 *N*-glycome after the treatment with different concentrations of 5-aza-2dC. Protein glycosylation is a compartmentalized, enzyme-directed process that involves complex dynamic interactions between hundreds of glyco-genes but also other, glycosylation-related genes. Our recent work has shown that elements of glycosylation (such as core fucosylation and/or glycan branching) are master regulated by certain transcription factors^40^. Knockout studies in mice have shown that there are some mechanisms of the redundancy of glycosyltransferases and compensation for the lack of the products of a certain glycosyltransferase^41^,^42^. Up- or down-regulation of only one glyco-gene may lead to complex changes in the *N*-glycan biosynthetic pathway and result in altered final glycan structure. On the other hand, a change in one glycosyltransferase can have a pleiotropic effect^43^. Therefore, it is not surprising that the composition of *N*-glycome of HepG2 secretome was differentially changed after the treatment with two different concentrations of 5-aza-2dC, reflecting the physiological status of a cell under conditions of different global genome methylation levels. Indeed, even the slightest change in genome methylation can result in perturbed physiological balance (cellular homeostasis) in a non-liner fashion, simultaneously affecting the expression of genes involved in many different biological processes, including protein glycosylation.

In the present study, we identified 18 out of 84 glyco-genes (involved in both *O*- and *N*-linked glycosylation) that were either down- or up-regulated as a result of DNA methylation change. We also performed a meta-analysis based on the publicly available expression and methylation datasets and identified the glyco-genes which most often show change in their expression and/or methylation level in different cancers, including HCC^44^. When we compared the glyco-genes from the present study that showed altered expression after the treatment with 5-aza-2dC with the glyco-genes found most noticeably changed in our meta-analysis, there was a considerable overlap. Eleven out of 18 glyco-genes from the present study were found within the identified genes with changed expression/methylation levels in different cancers, including HCC, from our meta-analysis. Moreover, the *MGAT3* gene appeared among the ten highest ranked genes according to changes in expression and methylation levels in different cancers. Therefore, we conducted a more detailed analysis of this gene in HCC and HepG2 cell line from the publicly available datasets and found that the *MGAT3* is hypomethylated and overexpressed in patients with HCC.

The most consistent glycosylation change resulting from the treatment with both concentrations of 5-aza-2dC was a decrease in highly branched glycan structures. This change could be put into relation with the increased expression of the *MGAT3* gene (Fig. 5a) which encodes for UDP-*N*-acetylglucosamine:β-D-mannoside β1–4 *N*¿acetylglucosaminyltransferase III (GnTIII). This enzyme transfers a GlcNAc residue to the beta-linked mannose of the 3-mannose core of *N*-linked oligosaccharides forming a unique structure, the bisecting GlcNAc. The addition of a bisecting GlcNAc inhibits elongation of the glycan structure by inhibiting GnT-IV, GnT-V and Fut8 (Fig. 5b), *i.e.* prevents core-fucosylation and branching^31^. We observed both glycan changes following the 5-aza-2dC treatment. Studies of overexpression of Gnt-III in transgenic mouse hepatocytes have described pathological mechanism in which an aberrant glycan structure (bisecting GlcNAc) can modify certain biochemical parameters in liver^45^. Also, it has been shown that overexpression of Gnt-III causes a decrease in the level of β1–6-branched *N*-glycans^36^,^46^.

Several recent studies on HCC patients or HCC corresponding cell lines have shown the same aberrant type of glycans either on cell surface glycoproteins, cell secretome or in plasma. For instance, core-fucosylated glycans increase on specific non-immunoglobulin proteins in both patients and some animal models of HCC^47^. On the other hand, two independent groups reported highly specific HCC glycoprotein markers, *i.e.* core-fucosylated tri- and tetra-antennary glycans, in the serum of HCC patients^32^,^48^. Alteration in transcription and translation levels of enzymes GnT-III and GnT-V were related to corresponding changes in highly branched glycan structures in HCC cell line as well^49^. In addition, branched glycan structures, structures with sialic acid and core fucosylated structures are found to be highly expressed in HCC and these changes were closely related to the transcriptional and translational levels of the glyco-genes^50^.

In our study, the *MGAT3* gene was up-regulated following the treatment with 5-aza-2dC, and at the same time the quantity of the highly branched glycans was decreased within the *N*-glycome of the HepG2 secretome. However, we did not detect an increase of bisecting GlcNAc. This could be due to the experimental limitations in measuring *N*-glycans, since glycans with bisecting GlcNAc were nearly undetectable in the HepG2 secretome. Furthermore, we measured only *N*-glycans from the secretome of HepG2 cells, and not *N*-glycans from glycoproteins on the cell surface. It has been shown that important target proteins for GnT-III are E-cadherin (when modified with GlcNAc, it is distributed to the cell surface) and integrin, the major cell surface glycoprotein^50^. Both the decrease in core-fucosylation (recorded after the treatment with 1 μM 5-aza-2dC) and the decrease in highly branched glycans (recorded after the treatment with both concentrations of 5-aza-2dC) are in concordance with the elevated expression level of the *MGAT3* gene due to decreased methylation level in the promoter/first intron of this gene. Interestingly, we did not observe changes in the expression of the *FUT8* gene (included in our gene expression array) after the 5-aza-2dC treatment. This gene encodes for α1,6-fucosyltransferase that catalyses the transfer of α1,6-fucose residue from GDP-fucose to the innermost GlcNAc residue of glycans on glycoproteins, *i.e.* it is responsible for core fucosylation (Fig. 5b). Anugraham and co-workers found that the bisecting GlcNAc was the most prominent change in the *N-*glycome of the cell membrane in several epithelial cancerous ovarian cell lines, and this change correlated with the increased expression level of the *MGAT3* gene; however, it did not correlate with the decreased expression of the *FUT8* gene even though lower levels of core-fucosylated glycans were detected^24^. Thus, it seems that a complicated interplay between *MGAT3*, *FUT8* and other glyco-genes, glycosylation-related genes and regulatory elements, as well as the intrinsic environment, regulates bisecting and core-fucosylation of *N*-glycans^42^.

In the current study, we propose that aberrant CpG methylation in the *MGAT3* gene promoter region could be one of the important mechanisms leading to aberrant protein glycosylation specific for HCC. Following 5-aza-2dC treatment that induced global genome DNA hypomethylation we were able to show that 22 out of 32 CpG sites in the promoter region and the first intron of the *MGAT3* gene changed methylation level. Even if we do not have direct evidence that methylation in some of these CpG sites has a regulatory role for the expression of this gene, the methylation level was significantly decreased and, at the same time, the *MGAT3* expression was significantly increased. In addition, our meta-analysis of the methylation/expression level of the *MGAT3* gene in 81 HCC patients and corresponding adjacent non-tumour tissue showed that this gene is hypomethylated in tumour and has increased expression. Interestingly, 10 (out of 32) CpG sites covered with 5 pyrosequencing assays did not undergo a change in the methylation level. This raises the question whether certain CpG sites are differentially affected by 5-aza-2dC treatment. It has been shown that differential sensitivity of particular loci/elements to re-methylation after the removal of DNMT inhibitor is determined in part by the histone code, present at these gene loci/elements before demethylation^51^.

## Material and Methods

### Cell culture preparation

HepG2 cells were cultured in Dulbecco’s modified Eagle’s medium (DMEM, Lonza), supplemented with 10% (v/v) heat inactivated foetal bovine serum (FBS, Gibco), 4 mM L-glutamine (Lonza), 100 U/mL penicillin and 100 μg/mL streptomycin (Lonza) in humidified incubator containing 5% CO_2_ at 37 °C. Cells were washed in phosphate buffered saline (PBS) and were detached with 0.25% (v/v) Trypsin-EDTA solution (Sigma-Aldrich).

### Treatment with 5-aza-2′-deoxycytidine

5-aza-2′-deoxycytidine (5-aza-2dC, Sigma-Aldrich) was dissolved in acetic acid:water (1:1) to 10 mM final concentration. 7.5 × 10^5^ cells were seeded in 10 cm and 3 × 10^5^ cells in 6 cm petri dishes. For immunofluorescence, cells were seeded on 13 mm coverslips. Twenty-four hours after seeding, the cells were incubated for 72 h with two different concentrations of 5-aza-2dC (1 μM and 2.5 μM). Every 24 h the medium was changed to a fresh one containing the same concentration of the inhibitor. Twenty-four hours before collection, cells were left in DMEM with 1 μM and 2.5 μM 5-aza-2dC and without FBS. Control cells were left untreated. This experimental setup was repeated at three time points. The first time point included three biological replicates of all groups while the second and the third time point consisted of five biological replicates for all groups.

### Immunofluorescence

For detection of 5-methylcytosine (5 mC), cells were fixed on coverslips with 3% paraformaldehyde (Sigma-Aldrich) for 5 min at room temperature and with ice-cold methanol for 5 min. Before and after the fixation cells were washed with PBS. Permeabilization was carried out using 0.5% Triton X-100 (Sigma-Aldrich) in PBS for 5 min at room temperature. DNA was denatured with 4M HCl for 10 min. Blocking was carried out in 4% BSA (Sigma-Aldrich) and 0.5% Triton X-100 in PBS for 30 min at room temperature. Cells were incubated with anti-5 mC antibody (1:200, Ab10805, Abcam) for 1 h and with FITC-labelled secondary antibody (1:100, Ab6785, Abcam) for 45 min. After counterstaining with DAPI (1 μg/mL, Sigma-Aldrich), the cells were mounted with ProLong Gold Antifade (Invitrogen) and analysed using an Olympus BX51 microscope. Fluorescence intensities were measured using the ImageJ software.

### CpG methylation analysis by pyrosequencing

For the quantitative measurement of DNA methylation level at specific CpG sites we developed assays for the *MGAT3* gene. For estimation of genome-wide methylation, we analysed the LINE-1 elements, since they comprise around 17% of the human genome. DNA was isolated from HepG2 cells using DNeasy Blood & Tissue kit (Qiagen). Five hundred nanograms of DNA was bisulphite converted using EZ-DNA Methylation Gold kit (Zymo Research). PCR reactions for LINE-1 elements and *MGAT3* were performed using PyroMark PCR Kit (Qiagen). Pyrosequencing assays were created according to the sequence of the *MGAT3* gene as available in the UCSC base (hg19, RefSeq NM_002409). Five pyrosequencing assays were developed for the *MGAT3* gene. The cycling protocol was as follows: initial polymerase activation step for 15 min at 95 °C, 50 cycles of 30 s denaturation at 95 °C, and primer annealing for 30 s at specific temperatures (Supplementary Table 3), followed by 30 s extension at 72 °C. A final extension was done for 10 min at 72 °C. CpG methylation was analysed by pyrosequencing using the PyroMark Q24 Advanced instrument (Qiagen) according to manufacturer’s instructions. The primers for PCR amplification and pyrosequencing primers for *MGAT3* gene are listed in Supplementary Table 3. Methylation levels were analysed at 18 CpG sites in the promoter region and at 14 CpG sites in the first intron of *MGAT3* gene (Fig. 7). Pyrosequencing assays for the LINE-1 element covered six CpG sites^39^. EpiTect PCR Control DNA Set (methylated and unmethylated human DNA, Qiagen) was used as control for both the PCR and the pyrosequencing reactions.

### Determination of HepG2 cell cycle profile following the treatment with two different concentrations of 5-aza-2dC

Untreated and treated HepG2 cells with 1 μM and 2.5 μM 5-aza-2dC (for 72 hours) were trypsinized and washed in PBS. After washing cells were resuspended in 1 mL of PBS and fixed with 3 mL of cold absolute ethanol. Ethanol was added slowly during vortexing of the cell suspension. Immediately prior to flow cytometry analysis, the cells were washed again in PBS and then treated with RNase A (20 μg/mL) for 30 minutes at 37 °C. After incubation the HepG2 cells were stained with propidium iodide (50 μg/ml) and analyzed on a Becton Dickinson FACS calibur flow cytometer (20,000 counts were measured). FlowJo software (TreeStar Inc.) was used to determine percentage of cells in each phase of the cell cycle (G1, S, G2/M). The analysis was performed on three biological replicates.

Percentages of the cells in each phase of the cell cycle were transformed using the arcsine square root transformation. We used Welch’s unequal variances *t*-test to compare each of the groups (cells treated with different concentrations of 5-aza-2dC) with the control group (untreated cells).

### Quantitative Reverse Transcription PCR (qRT-PCR)

RNA was isolated from cells using RNeasy Mini Kit (Qiagen). Reverse transcription of RNA was performed with RT^2^ First Strand Kit (Qiagen) according to the manufacturer’s protocol for usage of cDNA on Glycosylation RT^2^ Profiler PCR Array (Qiagen). qRT-PCR was performed on Glycosylation RT^2^ Profiler PCR Array (Qiagen), which includes assays for 84 glyco-genes, under the conditions recommended by the manufacturer.

qRT-PCR was performed in subsequent experiments using TaqMan^®^ probes for the *MGAT3* gene (Assay ID: Hs02379589_s1, Applied Biosystems) and for the *HMBS* gene (Assay ID: Hs00609297_m1, Applied Biosystems), according to the manufacturer’s protocol. For these reactions cDNA was prepared using the PrimeScript Reverse Transcriptase (Takara) and random hexamers (Applied Biosystems). Cycling conditions were as follows: 60 min at 42 °C and 15 min at 70 °C. Reactions were performed in Applied Biosystems 7300 and 7500 Fast Real-time PCR Systems (Applied Biosystems). For all experiments, relative quantification was performed using the comparative C_t_ method and the *HMBS* gene was used as endogenous control.

### Meta-analysis of promoter methylation and expression level of the *MGAT3* gene

Publicly available datasets for the *MGAT3* gene promoter methylation and expression level for this gene were downloaded from the GEO database^52^. Methylation datasets for HepG2 cell line and normal hepatocytes were found under the accession numbers GSM999338 and GSM999339, respectively. DNA methylation in hepatocellular carcinoma (HCC) and adjacent tissue was taken from the GSE54503 dataset (132 samples). Expression data for HCC was found under the accession numbers GSE60502 (18 patients with paired HCC and adjacent tissue) and GSE62232 (81 HCC patients and 10 normal liver samples). Differences in the *MGAT3* promoter methylation were visualized using the R language and environment for statistical computing (R Core Team, 2015) and the package “methyAnalysis”. Expression analysis was conducted using the R package “limma”.

### Glycan release and labelling

Before collection of glycoproteins, the cells were left to grow in DMEM without foetal bovine serum for twenty-four hours to avoid interference by medium protein glycosylation. Glycans from at least 50 μg of secreted proteins were released, labelled and purified as described previously^10^. Briefly, *N*-glycans were released by PNGase F (ProZyme), labelled with 2-aminobenzimidine (2-AB) fluorescent dye (Sigma-Aldrich) and purified by HILIC-SPE to remove excess free label.

### HILIC-UPLC

Fluorescently labelled *N*-glycans were separated by hydrophilic interaction liquid chromatography (HILIC) on a Waters Acquity ultra performance liquid chromatography (UPLC) instrument (Milford) consisting of a quaternary solvent manager, sample manager, column manager and a FLR fluorescence detector set with excitation and emission wavelengths of 330 and 420 nm, respectively. The instrument was under the control of the Empower 2 software, build 2145 (Waters, Milford). Labelled *N*-glycans were separated on a Waters BEH Glycan chromatography column, 100 × 2.1 mm i.d., 1.7 μm BEH particles, with 100 mM ammonium formate, pH 4.4, as mobile phase A and acetonitrile as mobile phase B. The separation method used a linear gradient of 80-55% acetonitrile (v/v) at the flow rate of 0.25 ml/min in a 60 min analytical run. Samples were maintained at 5 °C before injection and the column temperature was 60 °C. The system was calibrated using an external standard consisting of hydrolysed and 2-AB labelled glucose oligomers from which the retention times for the individual glycans were converted to glucose units (GU). Data processing was performed using an automatic processing method with a traditional integration algorithm after which each chromatogram was manually corrected to maintain the same intervals of integration for all samples. The chromatograms were separated in the same manner into 20 peaks and the amount of glycans in each peak was expressed as percentage of the total integrated area.

### MALDI-TOF-MS

Prior to MS analysis of each glycan peak, the fluorescently labelled HepG2 sample was fractionated by HILIC-UPLC on a Waters Acquity UPLC instrument. The separation was performed on a 100 × 2.1 mm i.d., 1.7 μm BEH column with 100 mM ammonium formate, pH 4.4 as solvent A, and acetonitrile as solvent B. The separation method used a linear gradient of 80–55% acetonitrile at the flow rate of 0.25 ml/min in a 60 min analytical run. Separated and collected fractions were dried using a vacuum concentrator (Thermo Scientific SpeedVac system, Thermo Fisher Scientific).

Dried HepG2 HILIC-UPLC fractions were reconstituted in 10 μL Milli-Q water maintained at ≥18.2 MΩ at 25 °C (MQ, Millipore), of which 2 μL was spotted on a MTP AnchorChip 600/384 TF MALDI target (Bruker Daltonics) and co-crystallized with 1 μL 5 mg/mL 2,5-dihydroxybenzoic acid (2,5-DHB, Bruker Daltonics) in 50% HPLC SupraGradient acetonitrile (ACN, Biosolve). Analysis was performed using an UltraFlextreme MALDI-TOF/TOF-MS equipped with a Smartbeam-II laser and controlled by flexControl 3.3 Build 108 (Bruker Daltonics).

Measurements were taken in linear positive mode (LP) (100 ns delay, 25 kV acceleration), linear negative mode (LN) (100 ns delay, 20 kV acceleration), reflectron positive mode (RP) (120 ns delay, 25 kV acceleration) and reflectron negative mode (RN) (120 ns delay, 20 kV acceleration). Before sample analysis, machine calibration was performed on a peptide calibration standard (Bruker Daltonics). Sample spectra were acquired within a window of *m/z* 1000 to 5000 for LN and LP modes, *m/z* 1000 to 4000 for RP mode and *m/z* 800 to 5000 for RN mode. Deflection was set below *m/z* 450 for the linear modes, and below *m/z* 600 for the reflectron modes. Of each sample, 5000 shots were summed using a laser repetition rate of 200 Hz.

### MALDI-TOF-MS data analysis

Data acquired by MALDI-TOF-MS was analysed using flexAnalysis 3.3 Build 65 (Bruker Daltonics) to reveal the most abundant glycan species in each HepG2 fraction. Spectra recorded for each fraction were analysed in LN, LP, RN and RP modes before reaching an assignment. Sialylated species were detected in negative modes as [M-H]^−^ with possible proton to sodium/potassium substitutions per sialic acid, while neutral glycans were predominantly detected in positive modes as [M + Na]^+^. As sialic acids show metastable decay when performing reflectron mode MALDI-TOF-MS, linear mode spectra were used to detect signals, with reflectron mode only serving for precise *m/z* determination of monoisotopic peaks. Using these monoisotopic peak masses, glycan compositions could be calculated, but usually with an uncertainty between sialylation and difucosylation residue masses (291.1 and 292.1 Da, respectively). Additional evidence for sialylation was provided by differences in analysed relative intensity between linear and reflectron modes, the presence of metastable peaks in reflectron modes and the pattern of neutral salt adduction.

### LC-ESI-MS(/MS)

Five microliters of the reconstituted HepG2 fractionations was further diluted to 55 μl in MQ, 13 μl of which was analysed by nano-reversed-phase-LC-ESI-ion trap-MS(/MS). Chromatographic separation was performed on an Ultimate 3000 RSLCnano system (Dionex/Thermo Scientific) with a 75 μm × 15 cm Acclaim Pepmap RSLC column (C18, 100 Å pore size, 2 μm particle size) and a 100 μm × 2 cm Acclaim Pepmap 100 trap column (C18, 100 Å pore size, 5 μm particle size) (both from Thermo Scientific). Solvents used were 0.1% formic acid (FA, Sigma-Aldrich) in MQ (A) and 95% acetonitrile in MQ (B) and all steps were performed at 32 °C. At a flow rate of 500 μl/min, the columns were equilibrated with solvent A for 5 min, after which the sample was loaded. Elution was performed as a linear gradient, starting at 0% solvent B (the rest being solvent A) and progressing to 25% solvent B in 15 min, followed to a 5 min gradient to 70% solvent B. This concentration was maintained for another 5 min, after which the column was brought to 0% solvent B in 1 min. Washing was performed with another 24 min of 0% solvent B.

Eluted samples were analysed with an AmaZon ion trap (Bruker Daltonics) in positive mode, using a nanospray source at 4500 V and performing evaporation at 250 °C with a dry gas flow of 5 l/min. MS spectra were recorded in a window of *m/z* 500–1600, with the five highest peaks automatically analysed by collision-induced dissociation MS/MS in a detection window of *m/z* 140–2200.

### LC-ESI-MS(/MS) data analysis

Data acquired from the LC-ESI-MS/MS was analysed for the varying HepG2 fractions using DataAnalysis 4.0 SP4 Build 281 (Bruker Daltonics). Glycan fragmentation was identified from the MS/MS spectra by screening for common [M + H]^+^ fragment ions (*m/z* 325.1 (Hex_2_), *m/z* 342.2 (2-AB-HexNAc_1_), *m/z* 366.1 (Hex_1_HexNAc_1_), *m/z* 407.2 (HexNAc_2_), *m/z* 487.2 (Hex_3_), *m/z* 512.2 (Hex_1_HexNAc_1_dHex_1_) and *m/z* 657.2 (Hex_1_HexNAc_1_Neu5Ac_1_). Compound MS/MS spectra were created for likely glycan precursors, and analysed for structurally informative fragmentation. Relative quantification of the glycans within a fraction was performed based on the cumulative signal intensities of all charge states found within a 2 min elution window. When contributing to more than 20% of the cumulative relative intensities, a glycan was considered major within a fraction.

Overall LC-ESI-MS/MS analysis showed more individual glycan species than MALDI-TOF-MS, as well as providing structural information, but the compositions detected were highly similar between both methods.

### Statistical analysis

All results are shown as mean value +/− standard deviation. Mann-Whitney U test was used to compare between the control and the treated groups. Since glycomics is a novel type of analysis and is more sensitive to slight variations in experimental procedure (sample collection, time period of measurements), additional care is needed to avoid false interpretation of results. As the experimental setup was repeated in three time points, association analyses between glycan levels and treatments were controlled for experimental variation by including time period of measuring as additional covariate in model. A linear mixed model was used for association analysis, where treatment was described as a fixed effect, while the time period of measuring was modelled as random effect. False discovery rate was controlled using the Benjamini-Hochberg procedure. Statistical significance was set at a *p* value of 0.05, and the effect size (r) was given where appropriate. Statistical analysis was performed using STATISTICA 12 (StatSoft, Inc., Tulsa, USA) and R (version 3.0.1).

## Additional Information

**How to cite this article**: Klasić, M. *et al.* DNA hypomethylation upregulates expression of the MGAT3 gene in HepG2 cells and leads to changes in N-glycosylation of secreted glycoproteins. *Sci. Rep.* **6**, 24363; doi: 10.1038/srep24363 (2016).

## Supplementary Material

Supplementary Information

## Figures and Tables

**Table 1:** Effect size (r) of the treatment with DNA methylation inhibitor on individual glycans from the HepG2 secretome.

Glycan	Treatment	r	*p*	Treatment	r	*p*
GP6	1	0.63	**0.006**	2.5	0.28	0.208
GP7	1	0.35	**0.018**	2.5	0.05	0.794
GP8	1	0.30	0.058	2.5	−0.10	0.622
GP9	1	0.19	0.268	2,5	0.02	0.923
GP10	1	0.25	0.146	2.5	−0.04	0.851
GP11	1	−0.06	0.718	2.5	0.01	0.957
GP12	1	−0.03	0.895	2,5	−0.37	**0.014**
GP13	1	0.13	0.164	2.5	−0.09	0.381
GP14	1	−0.03	0.895	2.5	−0.46	**0.005**
GP15	1	−0.04	0.718	2.5	0.18	**0.041**
GP16	1	−0.20	**0.002**	2.5	0.00	0.957
GP16a	1	0.03	0.890	2.5	0.17	0.174
GP17	1	−0.47	**0.014**	2.5	0.14	0.538
GP18	1	−0.41	**0.009**	2.5	0.07	0.738
GP19	1	−0.22	0.293	2.5	0.21	0.293
GP20	1	−0.72	**0.002**	2.5	−0.15	0.427
GP21	1	−0,11	0.709	2.5	0.19	0.427
GP22	1	−0.14	0.622	2.5	0.21	0.427
GP23	1	−0.40	0.087	2.5	−0.50	**0.029**
GP24	1	−0.54	0.058	2.5	−0.70	**0.009**

The treatment using 1 μM and 2.5 μM 5-aza-2dC resulted in changes in different glycan groups. The statistically significant *p*-values (after correction for multiple testing) are highlighted with bold letters.

## References

[b1] Dalziel M., Crispin M., Scanlan C. N., Zitzmann N. & Dwek R. A. Emerging principles for the therapeutic exploitation of glycosylation. Science 3343, 1235681-1–1235681-8 (2014). 24385630 10.1126/science.1235681

[b2] Moremen K. W., Tiemeyer M. & Nairn A. V. Vertebrate protein glycosylation: diversity, synthesis and function. Nat. Rev. Mol. Cell Biol. 13, 448–462 (2012). 22722607 10.1038/nrm3383 PMC3934011

[b3] Marth J. D. A unified vision of the building blocks of life. Nat. Cell Biol. 10, 1015–1016 (2008). 18758488 10.1038/ncb0908-1015 PMC2892900

[b4] Zoldoš V., Novokmet M., Bečeheli I. & Lauc G. Genomics and epigenomics of human glycome. Glycoconj. J. 30, 41–50 (2013). 22648057 10.1007/s10719-012-9397-y

[b5] Krištić J., Zoldoš V. & Lauc G. Complex genetics of protein glycosylation In Glycoscience: Biology and Medicine (eds Taniguchi N., Endo T., Hart G. W., Seeberger P. H. & Wong C. H.) Ch. 8, 1303–1310 (Springer Japan, 2015).

[b6] Nairn A. V. *et al.* Regulation of glycan structures in animal tissues: transcript profiling of glycan-related genes. J. Biol. Chem. 283, 17298–17313 (2008). 18411279 10.1074/jbc.M801964200 PMC2427342

[b7] Yamamoto-Hino M. *et al.* Identification of genes required for neural-specific glycosylation using functional genomics. PLos Genet. 6, e1001254 (2010). 21203496 10.1371/journal.pgen.1001254 PMC3009669

[b8] Lauc G. *et al.* Loci associated with *N*-glycosylation of human immunoglobulin G show pleiotropy with autoimmune diseases and haematological cancers. PLos Genet. 9, e1003225 (2013). 23382691 10.1371/journal.pgen.1003225 PMC3561084

[b9] Menni C. *et al.* Glycosylation of immunoglobulin G: role of genetic and epigenetic influences. PLos One 8, e82558 (2013). 24324808 10.1371/journal.pone.0082558 PMC3855797

[b10] Knežević A. *et al.* Variability, heritability and environmental determinants of human plasma *N*-glycome. J. Proteome Res. 8, 694–701 (2009). 19035662 10.1021/pr800737u

[b11] Knežević A. *et al.* Effects of aging, body mass index, plasma lipid profiles, and smoking on human plasma N-glycans. Glycobiology 20, 959–969 (2010). 20356825 10.1093/glycob/cwq051

[b12] Walt D. *et al.* Transforming Glycoscience: A Roadmap for the Future. Ch. 3, 37–55 (The Nacional Academies Press, 2012). 23270009

[b13] Stowell S. R., Ju T. & Cummings R. D. Protein glycosylation in cancer. Annu. Rev. Pathol. 10, 473–510 (2015). 25621663 10.1146/annurev-pathol-012414-040438 PMC4396820

[b14] Maverakis E. *et al.* Glycans in the immune system and the altered glycan theory of autoimmunity: a critical review. J. Autoimmun. 57, 1–13 (2015). 25578468 10.1016/j.jaut.2014.12.002 PMC4340844

[b15] Horvat T., Zoldoš V. & Lauc G. Evolutional and clinical implications of epigenetic regulation of protein glycosylation. Clin. Epigenetics 2, 425–432 (2011). 22704355 10.1007/s13148-011-0039-1 PMC3365393

[b16] Tsuboi S., Hatakeyama S., Ohyama C. & Fukuda M. Two opposing roles of O-glycans in tumor metastasis. Trends Mol. Med. 18, 224–232 (2012). 22425488 10.1016/j.molmed.2012.02.001 PMC3356160

[b17] Pucic M. *et al.* High throughput isolation and glycosylation analysis of IgG-variability and heritability of the IgG glycome in three isolated human populations. Mol. Cell Proteomics 10, M111. 010090 (2011). 21653738 10.1074/mcp.M111.010090 PMC3205872

[b18] Gornik O. *et al.* Stability of N-glycan profiles in human plasma. Glycobiology 19, 1547–1553 (2009). 19726492 10.1093/glycob/cwp134

[b19] Novokmet M. *et al.* Changes in IgG and total plasma protein glycomes in acute systemic inflammation. Sci. Rep. 4, 4347, 10.1038 (2014). 24614541 10.1038/srep04347 PMC3949295

[b20] Ladd-Acosta C. & Fallin M. D. The role of epigenetics in genetic and environmental epidemiology. Epigenomics, 10.2217/epi.15.102 (2015). 26505319

[b21] Lauc G., Vojta A. & Zoldoš V. Epigenetic regulation of glycosylation is quantum mechanics of biology. Biochim. Biophys. Acta 1840, 65–70 (2014). 23999089 10.1016/j.bbagen.2013.08.017

[b22] Horvat T., Mužinić A., Barišić D., Herak-Bosnar M. & Zoldoš V. Epigenetic modulation of HeLa cell membrane *N*-glycome. Biochim. Biophys. Acta 1820, 1412–1419 (2012). 22192783 10.1016/j.bbagen.2011.12.007

[b23] Horvat T. *et al.* Reversibility of membrane *N*-glycome of HeLa cells upon treatment with epigenetic inhibitors. PLos ONE 8, e54672 (2013). 23336012 10.1371/journal.pone.0054672 PMC3545996

[b24] Anugraham M. *et al.* Specific glycosylation of membrane proteins in epithelial ovarian cancer cell lines: Glycan structures reflect gene expression and DNA methylation status. Mol. Cell Proteomics 13, 2213–2232 (2014). 24855066 10.1074/mcp.M113.037085 PMC4159645

[b25] Saldova R. *et al.* 5-AZA-2′-deoxycytidine induced demethylation influences *N*-glycosylation of secreted glycoproteins in ovarian cancer. Epigenetics 6, 1362–1372 (2011). 22086115 10.4161/epi.6.11.17977

[b26] Pedersen M. E. *et al.* An epidermal microRNA regulates neuronal migration through control of the cellular glycosylation state. Science 341, 1404–1408 (2013). 24052309 10.1126/science.1242528

[b27] Bernardi C., Soffientini U., Piacente F. & Tonetti M. G. Effects of microRNAs on fucosyltransferase 8 (FUT8) expression in hepatocarcinoma cells. PLos ONE 8, e76540 (2013). 24130780 10.1371/journal.pone.0076540 PMC3793929

[b28] Agrawal P. *et al.* Mapping posttranscriptional regulation of the human glycome uncovers microRNA defining the glycocode. Proc. Natl. Acad. Sci. USA 111, 4338–4343 (2014). 24591635 10.1073/pnas.1321524111 PMC3964104

[b29] Kim Y. S. & Deng G. Aberrant expression of carbohydrate antigens in cancer: the role of genetic and epigenetic regulation. Gastroenterology 135, 305–309 (2008). 18558094 10.1053/j.gastro.2008.06.013

[b30] Pinho S. S. *et al.* E-cadherin and adherens-junctions stability in gastric carcinoma: functional implications of glycosyltransferases involving N-glycan branching biosynthesis, N-acetylglucosaminyltransferases III and V. Biochim. Biophys. Acta 1830, 2690–2700 (2013). 23671930 10.1016/j.bbagen.2012.10.021

[b31] Takahashi M., Kuroki Y., Ohtsubo K. & Taniguchi N. Core fucose and bisecting GlcNAc, the direct modifiers of the N-glycan core: their functions and target proteins. Carbohydr. Res. 344, 1387–1390 (2009). 19508951 10.1016/j.carres.2009.04.031

[b32] Kamiyama T. *et al.* Identification of novel serum biomarkers of hepatocellular carcinoma using glycomic analysis. Hepatology 57, 2314–2325 (2013). 23322672 10.1002/hep.26262

[b33] An H. J. *et al.* Profiling of glycans in serum for the discovery of potential biomarkers for ovarian cancer. J. Proteome Res. 5, 1626–1635 (2006). 16823970 10.1021/pr060010k

[b34] Kirmiz C. *et al.* A serum glycomics approach to breast cancer biomarkers. Mol. Cell Proteomics 6, 43–55 (2007). 16847285 10.1074/mcp.M600171-MCP200

[b35] Chen C. Y. *et al.* Fucosyltransferase 8 as a functional regulator of nonsmall cell lung cancer. Proc. Natl. Acad. Sci. USA 110, 630–635 (2013). 23267084 10.1073/pnas.1220425110 PMC3545778

[b36] Yoshimura M., Nishikawa A., Ihara Y., Taniguchi S. & Taniguchi N. Suppression of lung metastasis of B16 mouse melanoma by N-acetylglucosaminyltransferase III gene transfection. Proc. Natl. Acad. Sci. USA 92, 8754–8758 (1995). 7568011 10.1073/pnas.92.19.8754 PMC41045

[b37] Ohtsubo K., Chen M. Z., Olefsky J. M. & Marth J. D. Pathway to diabetes through attenuation of pancreatic beta cell glycosylation and glucose transport. Nat. Med. 17, 1067–1075 (2011). 21841783 10.1038/nm.2414 PMC3888087

[b38] Karahoca M. & Momparler R. L. Pharmacokinetic and pharmacodynamic analysis of 5-aza-2′-deoxycytidine (decitabine) in the design of its dose-schedule for cancer therapy. Clin Epigenetics 5, 3 (2013). 23369223 10.1186/1868-7083-5-3 PMC3570332

[b39] Nieto M. *et al.* The absence of p53 is critical for the induction of apoptosis by 5-aza-2′-deoxycytidine. Oncogene 23, 735–743 (2004). 14737108 10.1038/sj.onc.1207175

[b40] Zoldos V. *et al.* Epigenetic silencing of HNF1A associates with changes in the composition of the human plasma *N*-glycome. Epigenetics 7, 164–172 (2012). 22395466 10.4161/epi.7.2.18918 PMC3335910

[b41] Orr S. L. *et al.* A phenotype survey of 36 mutant mouse strains with gene-targeted defects in glycosyltransferases or glycan-binding proteins. Glycobiology 23, 363–380 (2013). 23118208 10.1093/glycob/cws150 PMC3605971

[b42] Kurimoto A. *et al.* The absence of core fucose up-regulates Gnt-III and Wnt target genes: a possible mechanism for an adaptive response in terms of glycan function. J. Biol. Chem. 289, 11704–11714 (2014). 24619415 10.1074/jbc.M113.502542 PMC4002080

[b43] Takamatsu S. *et al.* Physiological and glycomic characterization of *N*-acetylglucosaminyltransferase-IVa and–IVb double deficient mice. Glycobiology 20, 485–497 (2010). 20015870 10.1093/glycob/cwp200 PMC2900882

[b44] Vojta A., Samarzija I., Bockor L. & Zoldos V. Glyco-genes change expression in cancer through aberrant methylation. BBA Gen Subjects, 10.1016/j.bbagen.2016.01.002. 26794090

[b45] Ihara Y. *et al.* Ectopic expression of N-acetylglucosaminyltrasferase III in transgenic hepatocytes disrupts apolipoprotein B secretion and induces aberrant cellular morphology with lipid storage. Proc. Natl. Acad. Sci. USA 95, 2526–2530 (1998). 9482919 10.1073/pnas.95.5.2526 PMC19400

[b46] Koyota S. *et al.* Down-regulation of the alpha-Gal epitope expression in N-glycans of swine endothelial cells by transfection with the *N*-acetylglucosaminyltransferase III gene. Modulation of the biosynthesis of terminal structures by a bisecting GlcNAc. J. Biol. Chem. 276, 32867–32874 (2001). 11443114 10.1074/jbc.M102371200

[b47] Block T. M. *et al.* Use of targeted glycoproteomics to identify serum glycoproteins that correlate with liver cancer in woodchucks and humans. Proc. Natl. Acad. Sci. USA 102, 779–784 (2005). 15642945 10.1073/pnas.0408928102 PMC545516

[b48] Miyahara K. *et al.* Serum glycan as a prognostic marker in patients with advanced hepatocellular carcinoma treated with Sorafenib. Hepatology 59, 355–356 (2014). 23729393 10.1002/hep.26531

[b49] Liu T. *et al.* The transcriptional profiling of glycogenes associated with hepatocellular carcinoma metastasis. PLos ONE 9, e107941 (2014). 25232831 10.1371/journal.pone.0107941 PMC4169445

[b50] Zhao Y. *et al.* Branched *N*-glycans regulate the biological functions of integrins and cadherins. FEBS J. 275, 1939–1948 (2008). 18384383 10.1111/j.1742-4658.2008.06346.x

[b51] Kagey J. D., Kapoor-Vazirani P., McCabe M. T., Powell D. R. & Vertino P. M. Long-term stability of demethylation after transient exposure to 5-aza-2′-deoxycytidine correlates with sustained RNA polymerase II occupancy. Mol. Cancer Res. 8, 1048–1059 (2010). 20587535 10.1158/1541-7786.MCR-10-0189 PMC3086892

[b52] Barrett T. *et al.* NCBI GEO: archive for functional genomics data sets–update. Nucleic Acids Res. 41, D991–5, 10.1093/nar/gks1193 (2013). 23193258 PMC3531084

