# Supplementary material for: DNA hypomethylation upregulates expression of the MGAT3 gene in HepG2 cells and leads to changes in N-glycosylation of secreted glycoproteins
Source: Sci Rep. 2016 Apr 13;6:24363. doi: 10.1038/srep24363 (PMC4829869; doi:10.1038/srep24363)
Supplement: Supplementary Information [file srep24363-s1.pdf]

DNA hypomethylation upregulates expression of the *MGAT3* gene in HepG2 cells and leads to changes in *N*-glycosylation of secreted glycoproteins

Marija Klasić[1], Jasminka Krištić[2], Petra Korać[1], Tomislav Horvat[1], Dora Markulin[1], Aleksandar Vojta[1], Karli R. Reiding[3], Manfred Wuhrer[3,4], Gordan Lauc[2,5] , Vlatka Zoldoš[1,*]

[1]University of Zagreb Faculty of Science, Zagreb, Croatia

[2]Genos Glycoscience Research Laboratory, Zagreb, Croatia

[3]Center for Proteomics and Metabolomics, Leiden University Medical Center, Leiden, The Netherlands

[4]Division of BioAnalytical Chemistry, VU University Amsterdam, Amsterdam, The Netherlands

[5]University of Zagreb Faculty of Pharmacy and Biochemistry, Zagreb, Croatia

Corresponding author (*):

Vlatka Zoldoš

E-mail: vzoldos@biol.pmf.hr

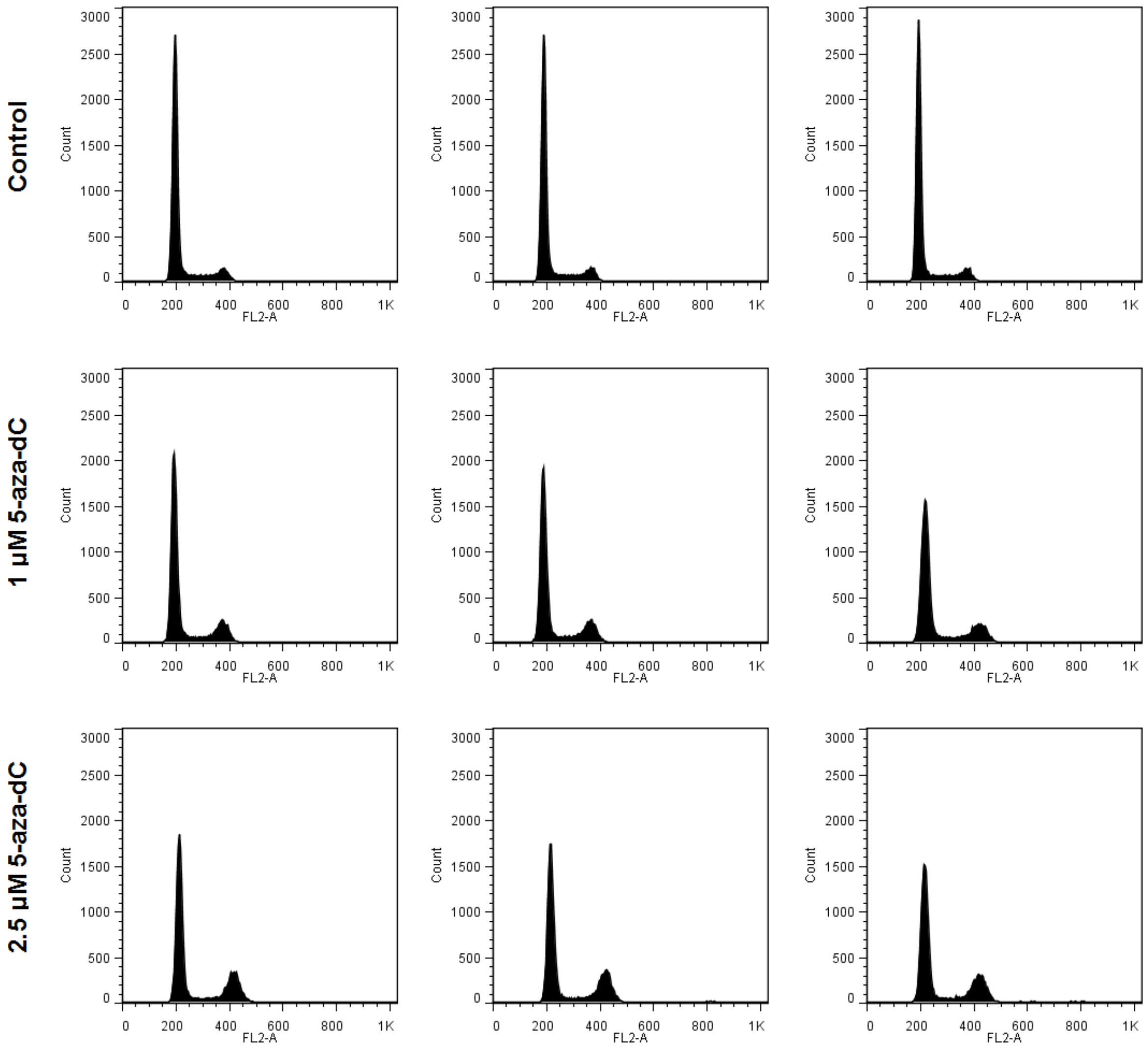

Supplementary figure 1. The cell cycle profiles of HepG2 cells cycle treated with 1 and 2.5 µM 5-aza-2dC. The cell cycle phase distribution was analysed using flow cytometry after 72h of the treatment. Both, treated and untreated cells were analysed in three biological replicates.

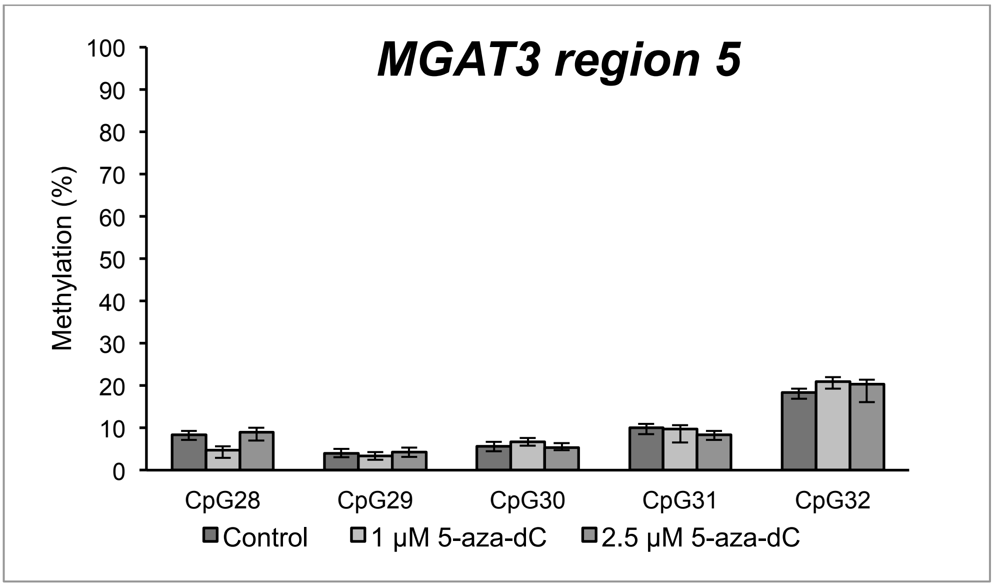

Supplementary Figure 2. Methylation level at five CpG sites in the *MGAT3* gene (the region 5, chr22: 39853538-39853827) was not significantly changed when compared the treated groups with the control group. Error bars show standard deviation between biological replicates.

Supplementary Table 1. Glycans detected per HepG2 LC fractionation by LC-MS(/MS) and MALDI-TOF-MS

Major species indicated in bold

LC-MS

fracti	precursor	charge	[M+H]	structure	composition	int.	rel. int
1				none			
2				none			
3				none			
4	678,34	2	1355,68	high M	**H5N2**	7057	1,00
5				poly H	cellulose/sepharose?		
6	725,95	3	2175,85	sulf aF	**H4N5F2 (SO_3)**	3705	0,26
6	840,36	2	1679,72	high M	**H7N2**	5916	0,42
6	925,37	2	1849,74	mix	**H5N3S1**	4600	0,32
7	840,35	2	1679,70	high M	**H7N2**	4084	1,00
8	685,33	3	2053,99	a+cF	**H5N4F2**	7563	0,23
8	773,97	3	2319,91	cF lnS ldn SO3	**H4N5S1F1-SO_3**	25473	0,77
9	684,98	3	2052,94	2a	**H5N4S1**	153312	0,89
9	773,98	3	2319,94	cF lnS ldnS SO3	H4N5S1F1-SO_3	19678	0,11
10	671,32	3	2011,96	mix	**H6N3S1**	134325	0,46
10	733,67	3	2199,01	cF	**H5N4S1F1**	159978	0,54
11	733,68	3	2199,04	aF	**H5N4S1F1**	110584	0,42
11	796,03	3	2386,09	a+cF ln/ldnS	**H4N5S1F2**	153295	0,58
11	733,98	3	2199,94	unlabeled aF mix	H7N3S1F1-unl		
12	782,36	3	2345,08	c+aF	**H5N4S1F2**	432957	0,95
12	843,67	3	2529,01	cF ldnS lnS	H4N5S2F1	23553	0,05
12	782,02	3	2344,06	unlabeled	H7N3S1F2-unl		
13	781,99	3	2343,97		**H5N4S2**	87491	0,47
13	844,35	3	2531,05	cF ln+ldnS	**H4N5S2F1**	72057	0,38
13	850,04	3	2548,12	a+cF 2a +N	H5N5S1F2	27851	0,15
13	693,65	3	2078,95	unlabeled	H5N4S1F1-unl		
14	830,69	3	2490,07	cF	**H5N4S2F1**	701475	1,00
14	790,66	3	2369,98	unlabeled	H5N4S2F1-unl		
15	879,35	3	2636,05	a+cF	**H5N4S2F2**	36779	1,00
16	904,05	3	2710,15	3a a+cF	**H6N5S1F2**	74664	1,00
16	742,33	3	2224,99	unlabeled	H5N4S1F2-unl		
16	804,35	3	2411,05	unlabeled lnS ldnS	H4N5S2F1-unl		
16a	904,05	3	2710,15	aF	**H6N5S1F2**	71202	1,00
16a	790,67	3	2370,01	unlabeled	H5N4S2F1-unl		
17	952,06	3	2854,18	3a a+cF	**H6N5S1F3**	66761	0,60
17	977,05	3	2929,15	4a cF	**H7N6S1F1**	26399	0,24
17	1001,04	3	3001,12	3a a+cF	H6N5S2F2	18476	0,17
17	790,67	3	2370,01	unlabeled	H5N4S2F1-unl		
18	952,73	3	2856,19	3a a+cF	**H6N5S1F3**	69770	1,00
18	839,36	3	2516,08	unlabeled	H5N4S2F2		
19	952,72	3	2856,16	a+cF	H6N5S1F3	7792	0,10
19	799,05	3	2395,15	4a aF	H7N6S1F1	7534	0,10

MALDI-TOF-MS negative

fracti	obs. mass	calc.mass	obs. - calc.	features	composition
1					
2					
3	1128,36				?
3	1186,34				?
3	1348,37			SO_3	?
4					
5					
6	1847,78	1847,67	0,11		H5N3S1
6	2172,97	2172,75	0,22	H2	**H4N5F2-SO_3**
7	1847,65	1847,67	-0,02		**H5N3S1**
7	2034,77	2034,75	0,02		**H4N4S1F1**
7	2091,81	2091,78	0,04		**H4N5S1**
7	2278,95	2278,86	0,09		**H3N6S1F1**
7	2358,94	2358,82	0,12	SO_3	H3N6S1F1-SO_3
8	2237,99	2237,83	0,15		**H4N5S1F1**
8	2317,95	2317,79	0,16	SO_3	H4N5S1F1-SO_3
9	2050,80	2050,75	0,05		**H5N4S1**
9	2237,95	2237,83	0,12		H5N5S1F1
9	2317,93	2317,79	0,14	SO_3	H4N5S1F1-SO_3
10	2009,95	2009,72	0,23		H6N3S1
10	2197,11	2196,81	0,30		**H5N4S1F1**
11	2197,03	2196,81	0,22		**H5N4S1F1**
11	2384,19	2383,89	0,30		**H4N5S1F2**
12	2197,10	2196,81	0,29		H5N4S1F1
12	2343,22	2342,86	0,36	F	**H5N4S1F2**
13	2051,05	2050,75	0,30		H5N4S1
13	2238,22	2237,83	0,39		H4N5S1F1
13	2342,11	2341,84	0,27	S	**H5N4S2**
13	2416,24	2415,88	0,35	HN	H6N5S1
13	2529,34	2528,93	0,41	S	**H4N5S2F1**
13	2546,37	2545,94	0,42		H5N5S1F2
14	1669,64	1669,62	0,02		H3N3S1F1
14	1831,69	1831,67	0,02		H4N3S1F1
14	2034,83	2034,75	0,08		H4N4S1F1
14	2180,97	2180,81	0,15	F	H4N4S1F2
14	2196,96	2196,81	0,16		**H5N4S1F1**
14	2488,01	2487,90	0,11	S	**H5N4S2F1**
14	2562,20	2561,94	0,26	HN	H6N5S1F1
15	2343,07	2342,86	0,20		H5N4S1F2
15	2489,25	2488,92	0,33	F	H5N4S1F3
15	2505,18	2504,92	0,26	H	H6N4S1F2
15	2562,20	2561,94	0,26		H6N5S1F1
15	2634,26	2633,96	0,30	S	**H5N4S2F2**
15	2708,30	2708,00	0,31	HNF	H6N5S1F2
15	2749,31	2749,02	0,28		H5N6S1F2
16	2197,06	2196,81	0,26		H5N4S1F1
16	2343,16	2342,86	0,30	F	H5N4S1F2
16	2562,34	2561,94	0,40	HN	H6N5S1F1
16	2708,40	2708,00	0,40	F	**H6N5S1F2**
16	2895,46	2895,08	0,38	F	H5N6S1F3
16a	2197,17	2196,81	0,36		H5N4S1F1
16a	2562,47	2561,94	0,53	HN	H6N5S1F1
16a	2708,53	2708,00	0,53	F	**H6N5S1F2**
16a	2781,51	2781,01	0,49		H7N6S1
17	2562,52	2561,94	0,58		H6N5S1F1
17	2707,58	2706,98	0,60		**H6N5S1F2**
17	2853,74	2853,03	0,71	SF	H6N5S2F1
17	2854,74	2854,06	0,69	SF	H6N5S1F3
17	2927,69	2927,07	0,62	HN	**H7H6S1F1**
17	2998,71	2998,07	0,64	S	**H6N5S3**
18	2708,37	2708,00	0,37		H6N5S1F2
18	2854,42	2854,06	0,37	F	**H6N5S1F3**
18	2927,45	2927,07	0,38		H7N6S1F1
19	2708,22	2708,00	0,22		**H6N5S1F2**
19	2854,31	2854,06	0,25	F	H6N5S1F3

MALDI-TOF-MS positive

fracti	obs. mass	calc.mass	obs. - calc.	features	composition	proton mass
1	1701,04	1701,55	-0,52	neg charge	**H4N4-SO_3**	
1	1881,13	1881,66	-0,53	neg charge	H4N3S1F2 - no AB	
1	2043,17	2043,71	-0,55	H neg	H5N3S1F2 - no AB	
1	2205,23	2205,77	-0,54	H2 neg	H6N3S1F2 - no AB	
2						
3	1188,46			neg charge	?	
3	1350,52			neg charge	?	
4	1377,54	1377,49	0,05	Na	**H5N2**	1355,537
5						
6	1701,72	1701,60	0,13	Na	**H7N2**	1679,724
6	1929,84	1929,71	0,13	Na	H5N4F1	1907,836
6	2116,93	2116,79	0,13	Na	**H4N5F2**	2094,926
7	1701,64	1701,60	0,04	Na	**H7N2**	1679,638
8	2075,81	2075,77	0,04	Na	**H5N4F2**	2053,805
8	2277,82	2277,82	0,00	Na NS	H5N5S1	2255,82
9	1783,70	1783,65	0,04	Na	H5N4	1761,695
9	1986,81	1986,73	0,08	Na N	H5N5	1964,805
9	2074,65	2074,75	-0,10	Na S	**H5N4S1**	2052,648
9	2277,75	2277,82	-0,07	Na NS	H5N5S1	2255,754
10	1863,72	1863,65	0,07	Na	H8N2	1841,717
10	1929,80	1929,71	0,10	Na	H5N4F1	1907,804
10	2220,86	2220,80	0,05	Na S	**H5N4S1F1**	2198,855
11	1929,95	1929,71	0,24	Na	H5N4F1	1907,952
11	2117,11	2116,79	0,31	Na	H4N5F2	2095,106
11	2221,03	2220,80	0,22	Na S	**H5N4S1F1**	2199,026
11	2408,16	2407,89	0,27	Na S	**H4N5S1F2**	2386,156
12	1929,84	1929,71	0,13	Na	H5N4F1	1907,837
12	2075,91	2075,77	0,14	Na	H5N4F2	2053,91
12	2366,97	2366,86	0,11	Na	**H5N4S1F2**	2344,97
13	2090,91	2090,72	0,19	K	H5N4S1	2052,912
13	2381,88	2381,81	0,07	K	**H5N4S2**	2343,88
13	2568,95	2568,90	0,05	K	**H4N5S2F1**	2530,95
14	1945,90	1945,68	0,22	K	H5N4F1	1907,903
14	2236,85	2236,78	0,07	K	H5N4S1F1	2198,852
14	2527,85	2527,87	-0,03	K	**H5N4S2F1**	2489,846
15	2674,05	2673,93	0,12	K	**H5N4S2F2**	2636,05
16	2456,99	2456,87	0,12	K	H6N5F2	2418,99
16	2748,07	2747,97	0,10	K	**H6N5S1F2**	2710,072
16a	2117,00	2116,77	0,23	K	H3N5F3	2079,001
16a	2407,97	2407,87	0,10	K	**H3N5S1F3**	2369,966
16a	2748,12	2747,97	0,15	K	**H6N5S1F2**	2710,119
17	2747,29	2747,97	-0,67	K	**H6N5S1F2**	2709,293
17	3039,15	3039,06	0,09	K	**H6N5S2F2**	3001,153
18	2603,15	2602,93	0,22	K	H6N5F3	2565,149
18	2894,08	2894,03	0,06	K	**H6N5S1F3**	2856,084
19	2748,23	2747,97	0,26	K	**H6N5S1F2**	2710,226
19	3038,98	3039,06	-0,08	K	**H6N5S2F2**	3000,983

**19**	**1001,05**	**3**	**3001,15**	**3a a+cF**	**H6N5S2F2**	**43904**	**0,56**
**19**	**1025,71**	**3**	**3075,13**		**H7N65S1F2**	**18842**	**0,24**
19	864,03	3	2590,09	unlabeled	H6N5S1F2-unl		
19	961,02	3	2881,06	unlabeled	H6N5S2F2-unl		
20	952,35	3	2855,05	cF	H6N5S2F1	10989	0,11
**20**	**1001,06**	**3**	**3001,18**	**a+cF**	**H6N5S2F2**	**65962**	**0,63**
**20**	**1025,75**	**3**	**3075,25**		**H7N6S1F2**	**27296**	**0,26**
20	912,35	3	2735,05	unlabeled	H6N5S2F1-unl		
21	904,36	3	2711,08	a+cF	H6N5S1F2	10837	0,09
21	1001,05	3	3001,15	a+cF	H6N5S2F2	19607	0,16
**21**	**1025,75**	**3**	**3075,25**	**later elute a+cF**	**H7N6S1F2**	**55880**	**0,45**
**21**	**1049,74**	**3**	**3147,22**	**a+cF**	**H6N5S2F3**	**38794**	**0,31**
22	904,02	3	2710,06	a+cF	H6N5S1F2	16560	0,19
**22**	**1074,44**	**3**	**3221,32**	**a+cF**	**H7N6S1F3**	**68450**	**0,81**
22	961,03	3	2881,09	unlabeled	H6N5S3-unl		
**23**	**842,69**	**4**	**3367,76**	**a+cF**	**H7N6S1F4**	**133278**	**0,51**
**23**	**878,86**	**4**	**3512,44**	**a+cF**	**H7N6S2F3**	**72696**	**0,28**
23	952,73	3	2856,19	a+cF	H6N5S1F3	34041	0,13
23	1001,03	3	3001,09	a+cF	H6N5S2F2	18941	0,07
23	961,07	3	2881,21	unlabeled	H6N5S3-unl		
**24**	**878,83**	**4**	**3512,32**	**a+cF**	**H7N6S2F3**	**63319**	**0,34**
**24**	**915,40**	**4**	**3658,60**	**a+cF**	**H7N6S2F4**	**122446**	**0,66**
24	757,31	4	3026,24	unlabeled	H6N5S3F1-unl		
24	793,84	4	3172,36	unlabeled	H6N5S3F2-unl		
24	830,33	4	3318,32	unlabeled	H6N5S3F3-unl		

**19**	2999,29	2999,09	0,20	S	**H6N5S2F2**
**19**	3073,29	3073,13	0,16	HN	H7N6S1F2
**20**	2708,51	2708,00	0,52		H6N5S1F2
**20**	2853,50	2853,03	0,46		H6N5S2F1
**20**	2854,50	2854,06	0,44		H6N5S1F3
**20**	2999,61	2999,09	0,51	SF	**H6N5S2F2**
**20**	3145,38	3145,15	0,23	F2S	H6N5S2F3
**21**	2708,34	2708,00	0,34		H6N5S1F2
**21**	2854,52	2854,06	0,47		H6N5S1F3
**21**	2927,47	2927,07	0,39		H7N6S1F1
**21**	2999,49	2999,09	0,40	S	H6N5S2F2
**21**	3073,42	3073,13	0,29	HNF	**H7N6S1F2**
**21**	3145,45	3145,15	0,30	FS	H6N5S2F3
**22**	2708,37	2708,00	0,37		H6N5S1F2
**22**	2853,98	2854,06	-0,07	F	H6N5S1F3
**22**	3000,59	3000,11	0,48	SF	H6N5S2F2
**22**	3219,41	3219,19	0,22	HNF	**H7N6S2F1**
**22**	3291,92	3292,20	-0,28	SF2	H6N5S3F2
**22**	3363,63	3363,20	0,42	HNF2	H7N6S2F2
**23**	2707,74	2708,00	-0,26		H6N5S1F2
**23**	2853,88	2854,06	-0,18	F	H6N5S1F3
**23**	3073,07	3073,13	-0,06	HN	H7N6S1F2
**23**	3219,29	3219,19	0,11	HNF	H7N6S1F3
**23**	3365,13	3365,25	-0,12	HNF2	**H7N6S1F4**
**23**	3509,91	3510,28	-0,37	HNSF	H7N6S2F3
**24**	2854,46	2854,06	0,40		H6N5S1F3
**24**	3219,41	3219,19	0,23	HN	H7N6S1F3
**24**	3365,27	3365,25	0,03	HNF	**H7N6S1F4**
**24**	3510,15	3510,28	-0,13	HNS	H7N6S2F3
**24**	3655,80	3656,34	-0,54	HNSF	**H7N6S2F4**

**20**	2748,21	2747,97	0,24	K	H6N5S1F2	2710,205
**20**	3039,27	3039,06	0,20	K	**H6N5S2F2**	3001,266
**21**	2806,07	2806,01	0,06	K	H6N6F3	2768,066
**21**	3113,06	3113,10	-0,04	K	**H7N6S1F2**	3075,056
**22**	2968,39	2968,06	0,33	K	H7N6F3	2930,393
**22**	3259,41	3259,16	0,25	K	**H7N6S1F3**	3221,409
**23**	3114,21	3114,12	0,09	K	H7N6F4	3076,213
**23**	3405,11	3405,22	-0,10	K	**H7N6S1F4**	3367,113
**24**						

Supplementary Table 2: Brief description of enzymes encoded by glyco-genes, which were down- or up-regulated after the treatment with 5-aza-2dC. Table provides information about the full name of an enzyme encoded by a certain glyco-gene, a short description of reaction catalyzed by this specific enzyme, a glycosylation pathway in which an enzyme is involved, and information about glycosylation feature/s which can be affected by a change in expression of a certain glyco-gene (Source: UniProt and KEGG).

**down-regulated glyco-genes**				
**glyco-gene**	**name of encoded enzyme**	**function of enzyme**	**type of glycosylation pathway**	**glycosylation feature/s affected by enzyme**
A4GNT	alpha-1,4-*N*-acetylglucosaminyltransferase	transfers GlcNAc to core 2 *O*-glycans	*O*-glycosylation	extension of *O*-glycans
B3GNT4	beta-1,3-N-acetylglucosaminyltransferase 4	transfers GlcNAc to glycolipid substrate and synthesizes poly-*N*-acetyllactosamine	glycosphingolipid glycosylation	poly-*N*-acetyllactosamine glycan extension
B3GNT8	beta-1,3-N-acetylglucosaminyltransferase 8	transfers GlcNAc to multiantennary glycans and synthesizes poly-*N*-acetyllactosamine	*N*-glycosylation<br>O-glycosylation	poly-*N*-acetyllactosamine glycan extension
GALNT14	polypeptide N-acetylgalactosaminyltransferase 14	transfers GalNAc to serine or threonine residue of protein	*O*-glycosylation	initial step of *O*-glycosylation
GALNT9	polypeptide *N*-acetylgalactosaminyltransferase 9	transfers GalNAc to serine or threonine residue of protein	*O*-glycosylation	initial step of *O*-glycosylation
GALNTL1	polypeptide N-acetylgalactosaminyltransferase-like protein 1	transfers GalNAc to serine or threonine residue of protein	*O*-glycosylation	initial step of *O*-glycosylation
GALNTL5	polypeptide *N*-acetylgalactosaminyltransferase-	transfers GalNAc to serine or threonine residue of protein	*O*-glycosylation	initial step of *O*-glycosylation

	like protein 5			
NEU4	sialidase 4	removes sialic acid residues from glycoproteins and glycolipids	*N*-glycosylation<br>glycosphingolipid glycosylation	sialylation
ST8SIA2	alpha-2,8-sialyltransferase 8B	transfers sialic acid to glycoproteins and can form polysialic acid structures	*N*-glycosylation	sialylation
ST8SIA6	alpha-2,8-sialyltransferase 8F	transfers sialic acid to glycoproteins and glycolipids, forms NeuAc-alpha-2,8-NeuAc structures, but not oligosialic or polysialic acid structures	*N*-glycosylation<br>O-glycosylation<br>glycosphingolipid glycosylation	sialylation
**up-regulated glyco-genes**				
**glyco-gene**	**name of encoded enzyme**	**function of enzyme**	**type of glycosylation pathway**	**glycosylation feature/s affected by enzyme**
GALNT12	polypeptide *N*-acetylgalactosaminyltransferase 12	transfers GalNAc to serine or threonine residue of protein	*O*-glycosylation	initial step of *O*-glycosylation
GALNT3	polypeptide *N*-acetylgalactosaminyltransferase 3	transfers GalNAc to serine or threonine residue of protein	*O*-glycosylation	initial step of *O*-glycosylation
GALNT8	polypeptide *N*-acetylgalactosaminyltransferase 8	transfers GalNAc to serine or threonine residue of protein	*O*-glycosylation	initial step of *O*-glycosylation
MGAT3	beta-1,4-mannosyl-glycoprotein beta-1,4-*N*-acetylglucosaminyltransferase	transfers bisecting GlcNAc to beta-linked mannose in core structure of *N*-glycoproteins	*N*-glycosylation	bisecting<br>branching<br>core-fucosylation

MAN1C1	mannosyl-oligosaccharide alpha-1,2-mannosidase IC	removes mannose residues from Man_9 structure and produces Man_8 structure	*N*-glycosylation	level of high-mannose glycans
MGAT2	alpha-1,6-mannosyl-glycoprotein beta-1,2-*N*-acetylglucosaminyltransferase	transfers GlcNAc to alpha-1,6 mannose in core structure of *N*-glycoproteins	*N*-glycosylation	synthesis of complex *N*-glycans
MGAT4C	alpha-1,3-mannosyl-glycoprotein beta-1,4-*N*-acetylglucosaminyltransferase C	transfers GlcNAc to alpha-1,3 mannose in core structure of *N*-glycoproteins and forms GlcNAc beta-1,4 branch	*N*-glycosylation	branching
ST8SIA3	alpha-2,8-sialyltransferase 8C	transfers sialic acid to glycoproteins and glycolipids; can form polysialic acid structures	*N*-glycosylation glycosphingolipid glycosylation	sialylation

Genes encoding enzymes involved in *N*-glycosylatio pathway are shaded in gray.
bisecting GlcNAc: GlcNAc that is inserted at the junction of two-branched (or highly branched) sugar chains
core structure of *N*-glycoproteins: consists of 3 mannose and 2 *N*-acetylglucosamine residues (Man3GlcNAc2)
GalNAc: *N*-Acetylgalactosamine
GlcNAc: *N*-Acetylglucosamine
poly-*N*-acetyllactosamine: chain consisted of repeating disaccharide GlcNac-galactose
polysialic acid: chain consisted of sialic acid residues

Supplementary Table 3. The primers and the annealing temperatures (Ta) for PCR amplification (Forward, F, and Reverse, R) and pyrosequencing (SEQ) of fragments in the promoter/first intron of *MGAT3* gene. Numbers 1-5 correspond to primers used for pyrosequencing the fragments within the regions 1 - 5.

Primer name	Sequence	Ta / °C
MGAT3F1	5'-GTTGGGATATAGAATAGGTAG-3'	54
MGAT3R1	5'-[Btn]ACCATTCCTCTCAAAACTCA-3'	
MGAT3F2	5'-GTTGTTGAGATTTAG-3'	51
MGAT3R2	5'-[Btn]CTAAAACTCTACCCTCC-3'	
MGAT3F3	5'-GTTTTTGAGTTTTGAGAGGAATGG-3'	60
MGAT3R3	5'-[Btn]ACCCTCTTAAACCTACTCTCCTAC-3'	
MGAT3F4	5'-AGTAGATATATAGATTTTGTAGA-3'	47
MGAT3R4	5'-[Btn]AATCTATACCTATATATACATAAAC-3'	
MGAT3F5	5'-GGGTTGGGGTGGGAGGTTTT-3'	60
MGAT3R5	5'-[Btn]CCCTCTCCACATTTACCTCTACCT-3'	
MGAT3_SEQ1	5'-GTTGGGATATAGAATAGGTAG-3'	
MGAT3_SEQ2	5'-GGTGAGTTGATTT-3'	
MGAT3_SEQ3	5'-GTTTTTGAGTTTTGAGAGGAATGG-3'	
MGAT3_SEQ4	5'-AGTAGATATATAGATTTTGTAGA-3'	
MGAT3_SEQ5	5'-GGGTTGGGGTGGGAGGTTTT-3'	
